# Health system strategies to increase HIV screening among pregnant women in Mesoamerica

**DOI:** 10.1186/s12963-018-0162-8

**Published:** 2018-03-20

**Authors:** Charbel El Bcheraoui, Paola Zúñiga-Brenes, Diego Ríos-Zertuche, Erin B. Palmisano, Claire R. McNellan, Sima S. Desai, Marielle C. Gagnier, Annie Haakenstad, Casey Johanns, Alexandra Schaefer, Bernardo Hernandez, Emma Iriarte, Ali H. Mokdad

**Affiliations:** 1 Institute for Health Metrics and Evaluation, 2301 5th Ave, Suite, Seattle, WA 600 USA; 2 Salud Mesoamérica 2015 / Inter-American Development Bank, Calle 50, Edificio Tower Financial Center (Towerbank), Piso 23, Panamá, Panamá

**Keywords:** Human immunodeficiency virus, Antenatal care, HIV screening, Health care disparities, Central America, Mesoamerica

## Abstract

**Background:**

To propose health system strategies to meeting the World Health Organization (WHO) recommendations on HIV screening through antenatal care (ANC) services, we assessed predictors of HIV screening, and simulated the impact of changes in these predictors on the probability of HIV screening in Guatemala, Honduras, Mexico (State of Chiapas), Nicaragua, Panama, and El Salvador.

**Methods:**

We interviewed a representative sample of women of reproductive age from the poorest Mesoamerican areas on ANC services, including HIV screening. We used a multivariate logistic regression model to examine correlates of HIV screening. First differences in expected probabilities of HIV screening were simulated for health system correlates that were associated with HIV screening.

**Results:**

Overall, 40.7% of women were screened for HIV during their last pregnancy through ANC. This rate was highest in El Salvador and lowest in Guatemala. The probability of HIV screening increased with education, household expenditure, the number of ANC visits, and the type of health care attendant of ANC visits. If all women were to be attended by a nurse, or a physician, and were to receive at least four ANC visits, the probability of HIV screening would increase by 12.5% to reach 45.8%.

**Conclusions:**

To meet WHO’s recommendations for HIV screening, special attention should be given to the poorest and least educated women to ensure health equity and progress toward an HIV-free generation. In parallel, health systems should be strengthened in terms of testing and human resources to ensure that every pregnant woman gets screened for HIV. A 12.5% increase in HIV screening would require a minimum of four ANC visits and an appropriate professional attendance of these visits.

## Background

The Mesoamerican region faced challenges working towards Millennium Development Goals 4 and 5 of reducing child and maternal mortality [1]. The Mesoamerican Health Initiative, Salud Mesoamérica (SMI), was launched in 2009, as a health system strengthening (HSS) strategy to address the health issues faced by the poorest quintile of the population in El Salvador, Guatemala, Honduras, Nicaragua, Belize, Costa Rica, Panama, and the State of Chiapas in Mexico [2–4]. This public-private partnership between eight Mesoamerican countries and several international donors works to deliver integrated supply- and demand-side interventions by using results-based financing, implementing evidence-based health policies and interventions, and creating incentives to increase the use and quality of maternal and child health services for the poorest quintile of the population. One of SMI’s adopted strategies is Essential Obstetric and Neonatal Care, which focuses on providing women with the best, evidence-based practices for their reproductive health [5].

The HIV epidemic is a health challenge in Mesoamerica, and socio-economic factors contribute to HIV disparities in the region, especially among the prenatally-infected populations [6, 7]. In 2015, HIV/AIDS, claimed more than 19,000 disability-adjusted life years from Mesoamerican children less than 5 years old living in El Salvador, Guatemala, Honduras, Mexico, Nicaragua, and Panama [7] with prevalence increasing among women of reproductive age since 2005, and in all six countries except for Guatemala. All Mesoamerican countries identify pregnant women among their HIV high-risk groups, given the potential of HIV transmission to children during pregnancy, and adopted policies for HIV-free generations by 2015 [8–19]. However, in 2014, all of Panama, Honduras, Guatemala, El Salvador, Mexico, and Nicaragua registered more than 1000 new HIV cases each [10–12, 17–19]. Mother-to-child transmission is responsible for 3.2%, 5.1%, 6.0%, and 6.1% of new cases in Panama, Guatemala, El Salvador, and Honduras, respectively (no relevant information is available for Mexico and Nicaragua) implying over 200 new infected babies based on the number of HIV cases for 2014, and making the HIV-free generation goals harder to achieve [20].

Given the continued mother-to-child transmission in these six countries, it is crucial to assess the stages of the HIV care cascade that need attention. In line with the World Health Organization guidelines for prevention of this mode of HIV transmission, pregnant women are to receive antenatal care services during which they need to be screened for HIV, and then linked to treatment in case they were positive, in order to prevent transmission to their baby [21, 22]. HIV screening through ANC is the first and most crucial step in preventing mother-to-child transmission. However, the rates and determinants of ANC and HIV screening in pregnancy in Mesoamerica are unknown in the absence of established surveillance systems to monitor these essential indicators, making it impossible to track countries’ progress on ANC and the goals of HIV-free generations. SMI is paralleled with a prospective evaluation process, based on household, health facility, and school surveys. We analyzed data from the first round of household surveys of the SMI in Guatemala, Honduras, Mexico, Nicaragua, Panama, and El Salvador to 1) assess predictors of low coverage of ANC and HIV screening, and 2) simulate the impact of modifiable predictors to improve rates of HIV screening among pregnant women.

## Methods

### Aim, design, and setting

The data analyzed were collected as part of SMI’s evaluation plan. In order to establish the baseline performance for SMI indicators, surveys were conducted in households and health facilities in each country. Governments and the Inter-American Development Bank worked together to identify the poorest geographic regions in each country, which were designated as the target areas for the initiative. Using the most recent national census, these areas were divided into segments designed to contain between 150 and 200 households. Small segments were grouped to meet a minimum sample size and, on few occasions, large areas were split using roads. The minimum sample size was reached based on power calculation for each of the indicators. To select study participants, we followed the following four steps:

First, we conducted our own censuses of all households within each selected primary sampling unit; this ensured we used the correct denominator in indicator estimation and allowed us to account for the potential movement of the poor population in the study areas since the last national census. Second, we identified households eligible for the survey and which constituted our sampling frame. These are households that have at least one woman 15–49 years of age, or one child under the age of 5. Third, from eligible households, a randomly selected subset was chosen for the survey. All households sampled at this stage completed a household questionnaire [4]. The household questionnaire collected information on the source of water, type of toilet facilities, exposure to secondhand smoke, ownership of various assets including durable goods, agricultural land, and livestock, and household expenses and sources of health care financing. Fourth, women of reproductive age in the selected households completed the maternal and child health questionnaire.

### Participants

The maternal and child health questionnaire collected information from all women of reproductive age (15–49 years) in the selected households. Women with children aged 0–5 years were asked detailed questions in reference to each child born in the past 5 years regarding recent illness, diet, and vaccination history, which was captured using both caregiver recall and vaccination card (when available). Women were also asked questions about different health-related topics including antenatal, delivery, and postpartum care and detailed questions in reference to each child born in the past 5 years on ANC, such as services received during their visits, including if they had received an HIV test as part of these visits. Since data were self-reported, we analyzed data from the most recent pregnancy to account for recall bias.

The SMI surveys were conducted using a computer-assisted personal interview. Field surveyors were trained to enter data directly into the computer while administering the survey to the respondents. Additional details on SMI methodology and implementation are available elsewhere [4].

### Statistical analysis

Backward elimination multivariable logistic regression models were developed for each of the following outcomes of interest: receiving ANC, being offered an HIV test, and having received an HIV test through ANC. Potential predictors of interest included age, marital status, education, household monthly expenditure, health care coverage, number of ANC visits, type of health care setting visited for ANC, attendant of ANC visits, and country of residence. For comparisons between countries, Guatemala was used as the reference country based on alphabetical order. Data were weighted to reflect 1) the probability of the segment being selected, 2) the probability of the household being selected, 3) the proportion of women surveyed in the selected households, and 4) the post-stratification differences in age and sex distribution between the samples and the census. We modeled the data using SAS 9.3 to account for the complex survey design.

Later, we simulated the distribution of the expected probability of HIV screening based on the regression model described above for factors that were significantly associated with HIV screening, and were modifiable. For our analysis, a modifiable factor is any factor that is not biologically or socially fixed and can be changed through policy or intervention. For example, sex and age are not modifiable factors, whereas having health insurance and receiving ANC services are. These factors were the number of ANC visits received during pregnancy, and the type of health care attendant during ANC visits. For example, we simulated the distribution of the expected probability of HIV screening if all women received 1) four or more, or 2) three or less NAC visits. Then, first differences were calculated as the differences between the means of these two distributions of expected probabilities. This method is explained in detail by King et al. 2000 [23]. For each factor, we simulated the expected probability for the best and the worst scenario, then computed the differences in the expected probabilities. For comparison purposes, we also repeated this analysis by women’s educational level. We used R to simulate the expected probabilities and first differences.

## Results

Between March 2011 and August 2013, a total of 26,172 women of reproductive age were interviewed from the six countries. Their sociodemographic characteristics are presented in Table 1.

**Table 1:** Sociodemographic characteristics of women of reproductive age interviewed

Sociodemographic factors	Categories	N^a^ (weighted %)
Age group (years)	15–19	5398 (21.23)
	20–29	10,351 (35.80)
	30–39	6771 (26.40)
	40–49	3647 (16.57)
Educational level	No education	4267 (15.05)
	Primary school or literacy course	10,817 (44.62)
	Secondary school	5608 (24.10)
	High school	2222 (10.26)
	Technical school or university	831 (5.97)
Marital status	Never married	6897 (30.60)
	Currently married or in a relationship	17,460 (62.79)
	Separated, divorced, or widowed	1691 (6.61)
Household economic level	Lowest monthly expenditure tertile	7103 (28.09)
	Middle monthly expenditure tertile	12,921 (49.71)
	Highest monthly expenditure tertile	6143 (22.19)
Country	Guatemala	5827 (6.16)
	Honduras	3536 (10.74)
	Mexico	6935 (50.42)
	Nicaragua	2810 (23.79)
	Panama	2349 (1.46)
	El Salvador	4710 (7.42)

^a^frequencies are not weighted

Of these 16,259 (62.1%), representing a weighted estimate of 400,115 women in the studies areas, delivered a live birth within the last 5 years.

Among Mesoamerican women who delivered a live birth within the last 5 years in the studies areas, we estimate that 374,295 (93.5%; 95% confidence interval [CI]: 92.7–94.4), or an unweighted frequency of 14,801 (91.0%), received ANC. This varied from as low as 80.2% (95% CI: 77.5–82.9) in Guatemala to almost universal coverage in El Salvador (98.0%; 95% CI: 97.4–98.6). Reception of antenatal care varied according to women’s educational level, marital status, household economic level, and health care coverage (Table 2). Educated and married women, those who lived in a household of middle and higher expenditure tertiles, and those who had insurance were more likely to have received ANC during their last pregnancy.

**Table 2:** Antenatal care reception during last pregnancy, Mesoamerican women, 15–49 years old

		Received antenatal care during last pregnancy		
Factor	Categories	N (weighted %); SE	AOR	95% CI
Age group (years)	15–19	1689 (94.11); 0.77	REF	
	20–29	7275 (93.90); 0.46	0.89	0.65–1.21
	30–39	4500 (93.94); 0.60	0.96	0.70–1.32
	40–49	1132 (91.74); 1.07	0.79	0.51–1.24
Educational level	No education	2257 (87.82); 0.98	REF	
	Primary school or literacy course	6569 (93.34); 0.55	1.68	1.36–2.05
	Secondary school	2941 (96.40); 0.48	2.82	1.96–3.98
	High school	1121 (95.53); 0.87	2.59	1.64–4.00
	Technical school or university	463 (98.56); 0.63	6.79	2.27–20.30
Marital status	Never married	1572 (92.64); 0.97	REF	
	Currently married or in a relationship	12,075 (94.06); 0.42	1.92	1.42–2.60
	Separated, divorced, or widowed	902 (91.57); 1.23	1.16	0.75–1.81
Household economic level	Lowest monthly expenditure tertile	4556 (91.33); 0.72	REF	
	Middle monthly expenditure tertile	7294 (94.48); 0.43	1.31	1.07–1.61
	Highest monthly expenditure tertile	2951 (95.38); 0.62	1.38	1.02–1.88
Has medical insurance	No	10,252 (93.11); 0.46	REF	
	Yes	4118 (94.54); 0.67	2.05	1.48–2.85
Country	Guatemala	3003 (80.23); 1.37	REF	
	Honduras	2147 (95.42); 0.63	4.72	3.33–6.67
	Mexico	4246 (93.16); 0.72	1.71	1.23–2.37
	Nicaragua	1752 (96.36); 0.59	5.32	3.68–7.68
	Panama	1250 (84.30); 2.18	1.10	0.75–1.60
	El Salvador	2403 (98.02); 0.30	7.74	4.76–12.58

*AOR* Adjusted Odds Ratio, *95% CI* 95% confidence interval, *REF* Reference group, *SE* Standard Error

Among women who received ANC, the probability of being offered an HIV test was 0.43 (95% CI: 0.40–0.47) and varied between groups. For instance, the lowest probability, 0.20, was among women who had health insurance. The highest probability, 0.84, was among women who lived in El Salvador. Education, marital status, household economic level, medical insurance, number of ANC visits, type of attendants at ANC visits, health care settings where ANC visits were received, and country of residence all were associated with the probability of being offered an HIV test through ANC (Table 3).

**Table 3:** Offering HIV screening through antenatal care, last pregnancy, Mesoamerican women, 15–49 years old

		HIV test offered during antenatal care visit		
Factor	Categories	N (weighted %); SE	AOR	95% CI
Age group (years)	15–19	708 (44.90); 2.60	REF	
	20–29	3194 (43.95); 2.13	1.04	0.82–1.33
	30–39	1914 (43.87); 2.06	1.24	0.95–1.63
	40–49	404 (34.09); 2.63	1.09	0.74–1.59
Educational level	No education	466 (25.73); 2.30	REF	
	Primary school or literacy course	2184 (35.45); 2.06	1.23	0.97–1.57
	Secondary school	1607 (53.26); 2.58	2.22	1.67–2.95
	High school	594 (51.91); 2.72	3.51	2.45–5.03
	Technical school or university	328 (72.61); 4.07	2.53	1.69–3.79
Marital status	Never married	939 (71.44); 2.10	REF	
	Currently married or in a relationship	4833 (39.72); 1.88	0.65	0.49–0.86
	Separated, divorced, or widowed	414 (44.56); 3.13	.71	0.47–1.08
Household economic level	Lowest monthly expenditure tertile	1371 (32.14); 2.39	REF	
	Middle monthly expenditure tertile	3186 (44.96); 2.00	1.47	1.22–1.77
	Highest monthly expenditure tertile	1738 (60.54); 2.15	2.22	1.68–2.94
Has medical insurance	No	5168 (61.19); 1.69	REF	
	Yes	905 (20.02); 1.60	1.21	0.87–1.67
No. of antenatal care visits^a^	1–3	549 (29.50); 2.20	REF	
	4+	5568 (46.02); 2.00	1.99	1.58–2.50
Attendant of antenatal care visit^b^	Other	1283 (22.23); 1.81	REF	
	Physician or nurse	5001 (50.04); 2.00	1.89	1.50–2.39
Type of health care settings where antenatal care visits were received	Public hospital	813 (54.79); 3.10	REF	
	Public health unit	2217 (62.43); 2.62	0.88	0.67–1.15
	Other public health care setting	1386 (31.78); 2.26	0.80	0.62–1.03
	Private hospital	134 (60.16); 5.88	1.11	0.60–2.06
	Other private health care setting	445 (68.46); 2.77	1.60	1.11–2.32
Country	Guatemala	289 (10.03); 1.11	REF	
	Honduras	1403 (66.02); 2.03	5.69	3.28–9.87
	Mexico	741 (17.70); 1.53	0.84	0.57–1.25
	Nicaragua	1396 (79.69); 2.08	16.30	11.88–22.36
	Panama	432 (43.37); 3.62	1.95	1.20–3.15
	El Salvador	2034 (84.21); 1.00	17.91	12.76–25.14

*AOR* Adjusted Odds Ratio, *95% CI* 95% confidence interval, *REF* Reference group, *SE* Standard Error

^a^Number of ANC visits was categorized into “1–3” and “4+” based on countries’ guidelines for minimum numbers of visits required

^b^The “other” category included auxiliary nurse, laboratory technician, pharmacist, community health worker, and traditional midwives

Among women who received ANC, 40.7% (95% CI: 37.1–44.3) were screened for HIV. The probability of having received an HIV test through ANC was higher among women who completed primary school or took a literacy course (adjusted odds ratio [AOR]: 1.51; 95% CI: 1.16–1.95), secondary school (AOR: 2.68; 95% CI: 2.01–3.56), high school (AOR: 4.07; 95% CI: 2.82–5.86), or went to a technical school or university (AOR: 2.83; 95% CI: 1.95–4.10), women from the second tertile (AOR: 1.37; 95% CI: 1.14–1.63) or third tertile (AOR: 1.89; 95% CI: 1.43–2.49) of household monthly expenditure, those who had four or more ANC visits (AOR: 2.10; 95% CI: 1.70–2.71), those who were seen by a physician or nurse during their ANC visit (AOR: 2.10; 95% CI: 1.70–2.61), and women who lived in Honduras (AOR: 2.77; 95% CI: 1.65–4.66), Nicaragua (AOR: 14.20; 95% CI: 10.27–19.63), Panama (AOR: 2.35; 95% CI: 1.44–3.83), and El Salvador (AOR: 21.10; 95% CI: 14.71–30.13), compared to women with no schooling, those from the lowest household monthly expenditure tertile, those who received fewer than four ANC visits, those who were seen by other types of health care providers, and women living in Guatemala. This probability decreased for currently married women (AOR: 0.65; 95% CI: 0.49–0.87), and those who went to another type of public health care setting (AOR: 0.73; 95% CI: 0.57–0.93), compared to never-married women, and those who went to a public hospital for ANC (Table 4).

**Table 4:** HIV screening through antenatal care, last pregnancy, Mesoamerican women, 15–49 years old

		HIV test performed during antenatal care visit		
Factor	Categories	N (weighted %); SE	AOR	95% CI
Age group (years)	15–19	677 (42.44); 2.59	REF	
	20–29	3012 (41.41); 2.01	1.00	0.80–1.25
	30–39	1788 (41.04); 1.94	1.22	0.95–1.57
	40–49	374 (31.94); 2.57	1.06	0.74–1.52
Educational level	No education	432 (22.48); 2.03	REF	
	Primary school or literacy course	1979 (32.91); 1.94	1.51	1.16–1.95
	Secondary school	1553 (51.30); 2.57	2.68	2.01–3.56
	High school	564 (49.22); 2.68	4.07	2.82–5.86
	Technical school or university	311 (68.40); 3.82	2.83	1.95–4.10
Marital status	Never married	881 (67.62); 2.22	REF	
	Currently married or in a relationship	4536 (37.30); 1.78	0.65	0.49–0.87
	Separated, divorced, or widowed	398 (41.54); 3.08	0.66	0.44–0.99
Household economic level	Lowest monthly expenditure tertile	1291 (30.54); 2.27	REF	
	Middle monthly expenditure tertile	2971 (42.15); 1.94	1.37	1.14–1.63
	Highest monthly expenditure tertile	1664 (56.97); 2.07	1.89	1.43–2.49
Has medical insurance	No	4843 (57.37); 1.65	REF	
	Yes	858 (18.95); 1.52	1.29	0.92–1.82
No. of antenatal care visits	1–3	500 (26.55); 2.06	REF	
	4+	5252 (43.49); 1.91	2.10	1.70–2.61
Attendant of antenatal care visit	Other	1205 (20.85); 1.74	REF	
	Physician or nurse	4712 (47.15); 1.90	2.02	1.57–2.59
Type of health care settings where antenatal care visits were received	Public hospital	779 (52.78); 3.04	REF	
	Public health unit	2121 (58.55); 2.53	0.77	0.59–0.99
	Other public health care setting	1310 (29.95); 2.12	0.73	0.57–0.93
	Private hospital	119 (55.04); 6.14	0.89	0.47–1.70
	Other private health care setting	393 (63.12); 3.11	1.45	0.99–2.14
Country	Guatemala	263 (9.00); 1.00	REF	
	Honduras	1248 (59.79); 2.07	2.77	1.65–4.66
	Mexico	688 (16.59); 1.47	0.86	0.56–1.31
	Nicaragua	1309 (74.89); 2.11	14.20	10.27–19.63
	Panama	424 (43.12); 6.66	2.35	1.44–3.83
	El Salvador	1994 (82.41); 1.16	21.10	14.71–30.13

*AOR* Adjusted Odds Ratio, *95% CI* 95% Confidence Interval, *REF* Reference group, *SE* Standard Error

**Fig. 1:**
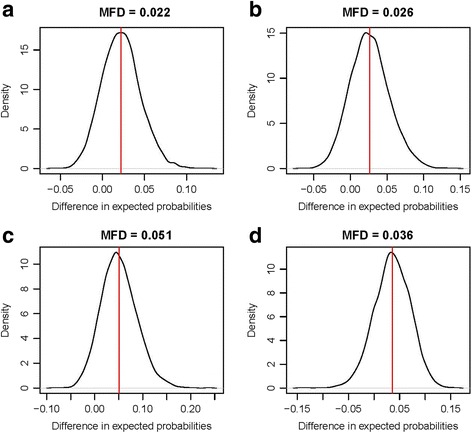
Distribution of differences in expected probabilities of HIV screening between (**a**) women who received four or more and those who received three or less antenatal care visits, (**b**) women who were attended by a physician or a nurse and those who were not, (**c**) women who received four or more antenatal care visits and were attended by a physician or a nurse, and those who received less than four antenatal care visits and were not attended by a physician or a nurse, and (**d**) most and least educated women (MFD: Mean first difference)Note: The area under the curve, in each panel, represents the distribution of the differences between the expected probabilities of HIV testing under two scenarios for a simulated modifiable factor. The red line represents the mean of the distribution. The x-axis represents the first differences in the expected probabilities of HIV screening and is limited to the probabilities obtained from the simulated first differences. The y-axis represents the density of the probability distribution of the first differences and is limited to the maximum observed density. For example, in panel A, the interval of the simulated first differences in the expected probabilities of HIV screening between women who received four or more and those who received three or less antenatal care visits is approximately [− 0.05 to 0.10]. However, the vast majority of this interval is [0.0 to 0.50], and the mean first difference is at 0.022

The probability of HIV screening among women who have received ANC based on the current data is at 0.41. If all women were to receive four or more ANC visits, their HIV screening probability would increase, on average, by 0.02 compared to the case where all women were to receive three or less ANC visits (Fig. 1a). HIV screening probability would increase, on average, by 0.026 if all women were to be seen by a physician or a nurse compared to the case where all women were seen by any other type of attendants (Fig. 1b). If all women were to receive at least four ANC visits and be seen by a physician or a nurse, their probability of being screened for HIV would increase by 0.05, bringing the current expected probability to 0.46 (Fig. 1c). That is 46% of pregnant women would be screened for HIV through ANC, a 12.5% increase from the current levels. If the educational level of all women would increase to the highest level as is currently in our sample, HIV screening probability would increase, on average, by 0.04, compared to the case where all women would still be at the lowest level of education as is in our sample (Fig. 1d).

## Discussion

We found large disparities, at the individual, institutional, and national levels, in provision of ANC services, and even larger ones in performing HIV screening during ANC when care was provided. While our study revealed a low rate of HIV screening during pregnancy in this population, it also proposed increasing the number of ANC visits and provision of care through ANC by nurses and physicians as a strategy to increase the probability of HIV screening during pregnancy to achieve HIV-free generations in the region. Our findings highlight the role of women’s education in receiving ANC and being screened for HIV. Increasing women’s educational level would, however, be a difficult intervention in a short time span. Married women were more likely to receive ANC than single women, but were less likely to receive HIV screening. Poverty also played a role in HIV screening rates. Women from the poorest households were the least likely to receive ANC, be offered an HIV test, and receive an HIV test. Those who had health coverage were more likely to receive ANC. At the institutional level, a higher number of ANC visits and the presence of a physician or a nurse increased the chances for HIV screening. When ANC was received through a smaller public health care facility (i.e., not a public health unit or hospital), women were more likely to be screened. Characteristics of health care facilities, such as staffing, bed size, or platform types, often have implications on clinical performance and patients’ health outcomes [24–26]. Beyond this, uptake on ANC and HIV screening during pregnancy varied widely between countries. While El Salvador achieved 98% coverage of ANC and an 84% HIV screening rate among pregnant women, only 80% of pregnant women received ANC in Guatemala. More so, only 9% of these were screened for HIV during their pregnancy. With these large disparities, achieving equity in health for all Mesoamerican women requires more focused efforts, and presents a challenge for SMI. Our most unfortunate finding is the fact that pregnant women in Panama, Honduras, and Guatemala were the least likely to be screened for HIV. In these countries, HIV/AIDS is the fifth-, third-, and eleventh-leading cause of death, respectively, for women of reproductive age.

Our study has several strengths as it is based on large sample sizes and we used standardized methods for data collection and strict measurements for data quality. It is also subject to some limitations. First, recall bias may have contributed to a lower reporting of testing. However, we restricted our analyses to the last pregnancy to reduce this effect. Also, it is possible that participants might be unaware of the tests they received due to their lack of knowledge around laboratory assessments, and providers might have followed an opt-out strategy for screening which was missed by our participants. This may be more relevant for women with less education. However, our data show low rates of screening among educated women. Further, our study did not collect information on the results of HIV screening from those who were tested. Since the aims of the data collection were not specific to HIV, we did not have IRB approval to collect this information. Finally, our study was conducted in the poorest areas and should not be representative of the whole country. However, all countries have the same health and preventive guidelines for the whole country.

Given that most of the published literature on HIV during pregnancy is from Africa, it is hard to compare our study to other ones. This is the first and largest comparative study to report on ANC and HIV screening during pregnancy in poor areas of six Mesoamerican countries, and propose health system solutions to increase the probability of HIV screening among pregnant women. More so, most of the published literature on this topic is restricted to identifying factors associated with HIV screening. Our study not only uncovers these but also measures the impact of their change on the probability of HIV screening.

To achieve an HIV-free generation, all pregnant women should be tested for HIV, and those who are found positive should receive therapy to ensure suppression of the virus and prevention of mother-to-child transmission [27]. Indeed, as HIV treatment has improved remarkably over the last decade, the chance of mother-to-child transmission can be reduced to less than 2% [28]. However, only women with known HIV status can receive the appropriate preventive protocol, underscoring the importance of HIV screening during pregnancy and labor. The Centers for Disease Control and Prevention recommends an opt-out policy for HIV screening of all pregnant women through ANC, as such a policy has proven to increase HIV screening rates for pregnant women compared to opt-in strategies [29]. Interestingly, women’s education has been documented as an essential factor in reducing child mortality [30]. This impact starts with ANC seeking and recognizing the importance of screening for HIV in preventing mother-to-child transmission. Our study shows that increasing women’s educational level can increase their probability of being screened for HIV during pregnancy. However, recommendations around this topic would be outside of the scope of our manuscript. Nevertheless, we found a serious deficit in the application of HIV screening during pregnancy by the medical systems in most of the countries we studied. HIV infection may be passed to women from their husbands who might be having extramarital sexual activities [31]. Hence, the importance of screening all pregnant women for HIV irrespective of their marital status should be enforced. It is possible that being married is perceived as a protective factor from HIV infection. However, in three of the studied countries (Honduras, Nicaragua, and El Salvador), heterosexual sex is the predominant mode of HIV transmission [8, 10, 13]. In Mexico and Panama, heterosexual and homosexual sex are equally responsible for HIV transmission [9, 11]. Therefore, no woman should be considered completely free of risk for HIV infection, even if married. Moreover, all countries’ guidelines for HIV screening do not include a separate protocol for married women who may opt-out from screening to avoid prejudice which is not in their, nor in their child’s, best interest.

Economic status is often a barrier or a facilitator to health care access, and Mesoamerican women, in our study, were impeded by their low economic status. The cost of health care services, whether direct, or indirect such transportation cost or travel time, can often be the biggest barrier [32]. When not sick, individuals might opt-out of health care services that are preventive in nature, such as ANC services, and thereafter might not be screened for HIV during pregnancy. On top of that, cultural beliefs are known to play a large role in people’s decision to use health care services or not. However, to reduce child mortality and improve maternal health, prevention should be given first priority, and preventive services should be accessible to everyone who chooses to use them. Hence, it is crucial to educate women, and the public in general, about the importance of HIV screening as a preventive measure for women and their unborn and newly born children.

Mesoamerican countries should consider an array of system improvements to ensure that all pregnant women receive ANC. Health promotion campaigns to increase awareness about maternal health should target the least educated and the poorest women. HSS strategies in Mesoamerica can now focus on two new areas to increase the rate of HIV screening among pregnant women and decrease the probability of mother-to-child transmission: 1) ensuring that every pregnant woman receives at least four ANC visits, and 2) be seen by a nurse or a physician; another option here would be to consider task-shifting where non-professional staff can be trained to apply rapid HIV-screening. To further benefit from ANC services, and reduce the chance for missed opportunities, we recommend screening pregnant women for HIV from the first ANC visit or the first contact with the health system during pregnancy. In countries where ANC is not optimal, countries should design continuous outreach campaigns that ensure that every pregnant woman is connected to care, especially in rural areas. Outreach health workers could either send culturally appropriate reminders about upcoming ANC visits via text message or provide reminders in person for women who do not own a cellular phone. Mobile clinics are another way to reach women in remote areas.

## Conclusions

Our study is of great importance to public health in Mesoamerica and the region as it draws attention towards the poorest and least educated women for ANC and HIV screening to ensure an HIV-free generation. If this segment of the population is not reached, it will slow national progress toward HIV-related objectives. Therefore, countries should work together to address these shortages in meeting WHO recommendations both for ANC and HIV screening during pregnancy. Countries with high uptake of these services should maintain their efforts, while countries that are lagging behind should change their strategies to ensure that every woman gets the ANC services she needs to ensure a healthier pregnancy and reduce the risk of HIV transmission to her child. Lessons learned should be shared in order to expedite improvements all over Mesoamerica.

Our study also calls for further investigation to close the gap in uncovering the remaining factors that can ensure an HIV screening of 100% among pregnant women in Mesoamerica. In the near future, we will prospectively assess the improvement resulting from SMI, specifically whether ANC services have improved in general, and if HIV screening during pregnancy has increased.

## Abbreviations

ANC: Ante-natal care
HIV: Human immunodeficiency virus
SMI: Salud mesoamerica initiative
